# Estetrol Increases Progesterone Genetic Response without Triggering Common Estrogenic Effects in Endometriotic Cell Lines and Primary Cultures

**DOI:** 10.3390/biomedicines11041169

**Published:** 2023-04-13

**Authors:** Daniel Patiño-García, Jaime Palomino, Cristián Pomés, Claudia Celle, Verónica Torres-Estay, Renán Orellana

**Affiliations:** 1 Departamento de Ciencias Químicas y Biológicas, Facultad de Ciencias de la Salud, Universidad Bernardo O’Higgins, General Gana 1702, Santiago 8370874, Chile; dafpaga@gmail.com (D.P.-G.); veronica.torres.e@gmail.com (V.T.-E.); 2 División de Ginecología, Escuela de Medicina, Pontificia Universidad Católica de Chile, Alameda 340, Santiago 8331150, Chile; cpomesc@gmail.com (C.P.); claudiacelle@gmail.com (C.C.); 3 Escuela de Medicina Veterinaria, Facultad de Ciencias Médicas, Universidad Bernardo O’Higgins, General Gana 1702, Santiago 8370874, Chile; jaime.palomino@ubo.cl; 4 Escuela de Química y Farmacia, Facultad de Medicina y Ciencia, Universidad San Sebastián, Bellavista 7, Santiago 8420524, Chile; 5 Programa de Magíster en Ciencias Químico Biológicas, Facultad de Ciencias de la Salud, Universidad Bernardo O’Higgins, General Gana 1702, Santiago 8370874, Chile

**Keywords:** endometriosis, estetrol, progesterone, resistance, hormones

## Abstract

Estetrol (E4), a natural estrogen produced by the human fetal liver, is actively studied for menopause and breast cancer treatment. It has low side effects and preferential estrogen receptor alpha (ER*α*) affinity. There are no data about its effects on endometriosis, a common gynecological disease affecting 6–10% of cycling women, generating painful pelvic lesions and infertility. Current combined hormone treatment (progestins and estrogens) is safe and efficient; nevertheless, one-third of patients develop progesterone (P4) resistance and recurrence by reducing P4 receptors (PRs) levels. We aimed to compare E4 and 17*β*-estradiol (E2) effects using two human endometriotic cell lines (epithelial 11Z and stromal Hs832 cells) and primary cultures from endometriotic patients. We evaluated cell growth (MTS), migration (wound assay), hormone receptors levels (Western blot), and P4 response by PCR array. Compared to E2, E4 did not affect cell growth or migration but increased estrogen receptor alpha (ER*α*) and PRs, and reduced ER*β*. Finally, the incubation with E4 improved the P4 gene response. In conclusion, E4 increased PRs levels and genetic response without inducing cell growth or migration. These results suggest that E4 might be useful for endometriosis treatment avoiding P4 resistance; however, evaluating its response in more complex models is required.

## 1. Introduction

Estetrol (E4) is a natural estrogen produced exclusively during pregnancy by the human fetal liver [1]. Binding studies revealed that E4 has a moderate but selective affinity for estrogen receptors (ERs), with a four-times higher preference for estrogen receptor alpha (ER*α*) over estrogen receptor beta (ER*β*) [2]. This resembles the activity of selective estrogen receptor modulators (SERMs), showing pro and anti-estrogenic effects. In some tissues, such as the brain [3,4], endothelium [5], bone [6], uterus [7], and ovary [8,9], E4 showed a behavior similar to 17*β*-estradiol (E2). On the other hand, anti-estrogenic effects of E4 were reported in the central nervous system [4,5] and epithelial breast cells [10,11]. This hormone has a high dose-related bioavailability and a long elimination half-life in human, allowing an oral daily use [8,12,13,14]; for these reasons, it is currently being investigated in clinical studies as a hormonal treatment for menopause among other diseases [9,15,16]. E4 was considered as a “weak” estrogen, representing a good candidate for an estrogen-dependent disease such as endometriosis; however, to date, there are no data about E4 effects in this pathology.

Endometriosis is one of the most common gynecological disorders. Around 6–10% of women of reproductive age (from the beginning of menstruation to menopause) in the western world are affected by this disease [17,18]. This percentage rises up to 50% in the infertile population [19] and to 60% in patients with chronic pelvic pain [20]. Endometriosis is defined as the presence of endometrial tissue (glands and stroma) outside the uterus. Due to E2 effects during each menstrual cycle, this tissue is able to proliferate developing lesions [21,22]. On the other hand, progesterone (P4) promotes endometriotic tissue atrophy, apoptosis, and autophagy [23,24,25]; therefore, current medical treatments are focused on stimulating P4 response or lowering E2 effects [26,27]. Synthetic versions of P4 (progestins) represent a safe option by reducing E2 levels [28] through its inactivation to estrone [29], and induce direct anti-proliferative and anti-inflammatory effects [30]. The combined hormone treatment of progestin and low doses of estrogen showed efficacy in pain relief and lower side effects with respect to progestin alone treatment [31]. However, around one third of patients developed “progesterone resistance”, which lead to symptoms reappearing and recurrent surgery [25,32,33]. Currently, a global reduction in progesterone receptors (PRs) is the most accepted hypothesis for developing resistance to treatment; however, the underlying mechanisms for this process remain unclear. To date, there is a clinical demand for an endometriosis treatment that does not develop P4 resistance.

A clear difference among eutopic and ectopic endometrium is their response to hormonal stimulus. Eutopic endometrium is sensitive to E2 during proliferative phase (due to increased levels of ERα); later, increments of PRs lead to P4 response during secretory phase [34,35]. On the contrary, endometriotic tissue is characterized by high levels of ERβ, and low amounts of ERα and PRs [36]; therefore, this tissue became highly sensitive to E2 and resistant to P4 effect. In endometriosis, ERβ is the main receptor involved in lesion development [37,38,39], and also can act as a transcriptional repressor of ERα [40]. Thus, permanent estrogenic stimulus results in high ERβ activity and low levels of ERα. Moreover, ERα activation is associated with PRs increment in endometrial tissue [36], but its role in endometriosis remains uncertain.

Considering its safety [13,14,15], E4 might represent a new hormonal therapeutic option for endometriosis, by activating Erα, it could increase PRs levels and restore P4 sensitivity. Therefore, the aim of this work was to evaluate, in vitro, the E4 effects in cell growth, migration, ERs and PRs levels using human epithelial and stromal cell lines and primary cultures.

## 2. Materials and Methods

### 2.1. Chemicals and Antibodies

First, 17*β*-estradiol, estetrol, and dimethyl sulfoxide (DMSO) were purchased from Sigma-Aldrich (St. Louis, MO, USA). Ethanol was acquired from Winkler (Santiago, Chile). The primary antibodies were PARP 1/2 (sc-7150) and ERα (F-10) (Santa Cruz, CA, USA). Progesterone receptor (6A1) (Cell Signaling, MA, USA), ERβ (Abx121395), and β-actin (Abx133823) (Abbexa, Cambridge, UK).

### 2.2. Study Ethical Approval

All experimental procedures were approved by the Ethical Scientific Committee at the Pontificia Universidad Católica de Chile N°170529004 and were endorsed by the Chilean National Fund of Science and Technology. All samples were collected at the Clinical Hospital of Pontificia Universidad Católica de Chile after obtaining written informed consent from the patients. By signing the informed consent, patients agreed to publish obtained results. All research was performed according to the approved guidelines and regulations.

### 2.3. Isolation of Primary Human Endometrial Stromal and Epithelial Cells

Endometrial biopsies were obtained from patients diagnosed with endometriosis who underwent laparoscopic surgery. Endometrial biopsies were taken using a sterile pipelle cannula. The protocol used was based on the previously described method [41]. Immediately after the collection of the endometrial biopsy, the tissue was rinsed with phosphate-buffered saline (PBS) to remove all blood and mucus.

The biopsy was cut into small fragments and incubated in phenol-red free DMEM/F12 supplemented with 0.5 mg/mL collagenase type I (Sigma-Aldrich, Overijse, Belgium) and supplemented with DNase I (0.1 mg/mL; Sigma-Aldrich, Overijse, Belgium) at 37 °C with agitation for 60 minutes on a shaker. After incubation, the resultant cell mixture was shaken thoroughly and poured through a 40 µm cell strainer. The filtrate containing the endometrial stromal cells (hESC) was centrifuged for 5 min at 720× *g*, resuspended in growth medium containing DMEM/F12 supplemented with 10% fetal bovine serum (FBS), 0.2% gentamicin, and 0.2% amphotericin B, and seeded in T75 flasks, whereas the endometrial epithelial cells (hEEC) were retained by the strainer. The epithelial fraction was collected by backwashing the cell strainer with 15 mL of growth medium consisting of DMEM/F12 supplemented with 10% FBS, 0.2% gentamicin, and 0.2% amphotericin B, and was centrifuged at 280× *g* for 5 min at room temperature. Then, the pellet was resuspended in 1 mL 0.25% trypsin-EDTA and incubated for 10 min at 37 °C to dissociate any epithelial clumps. Next, 9 mL of growth medium was added to mechanically dissociate any remnant of epithelial clumps by vigorously pipetting the solution up and down. Finally, solution containing hEEC was centrifuged at 280× *g* for 5 min at room temperature, and pellet was resuspended and seeded in T75 flasks. Cells were kept at 37 °C in 5% CO_2_ and the medium was changed every 2 days.

### 2.4. Cell Lines

Stromal endometriotic cell line Hs832 was acquired in ATCC (Manassas, VA, USA) and epithelial endometriotic cell line 11Z [42] was kindly provided by Dr. Gareth Owen (Pontificia Universidad Católica de Chile). All cell lines were Mycoplasma-free and maintained in phenol-red DMEM/F12 10% FBS, cultured in standard tissue plates until 80% confluence, and then, medium was replaced and supplemented with 5% charcoal-stripped FBS overnight (12 h). Incubation with physiological and supraphysiological concentrations of E2 (1 × 10^−11^ to 1 × 10^−7^ M) [43] or E4 (1 × 10^−9^ to 1 × 10^−5^ M) [44] was performed separately for 24 h. Ethanol and DMSO were used as the respective vehicle controls.

### 2.5. Cell Viability Assay

Cells were plated into 96-well plates with fresh phenol-red free DMEM/F12 5% charcoal after E2 and E4 treatments for 24 h, 5 × 10^−8^ M estradiol, 1 × 10^−8^ M BSA-conjugated estradiol, and 1 × 10^−8^ M G-1 for 4–18 h. Ethanol, phosphate-buffered saline (PBS), and DMSO were used as the respective vehicle controls, 15 μL of MTS solution was added at 37 °C for 2 h and was read using photometric reading [45].

### 2.6. Migration Assay

11Z and Hs832 cells were cultured into 6 well plates until 80% confluence, and then, a vertical wound (scratch) was introduced through the cell monolayer using a fine pipette tip. Medium was replaced with fresh DMEM/F12 containing 5% charcoal treated serum with E2 or E4. Wound closure was assessed by photography at 12, 24, and 48 h and quantified with Image J software.

### 2.7. RNA Isolation and RTq-PCR

Total RNA was isolated using GenElute^TM^ Mammalian Total RNA Miniprep Kit (Sigma-Aldrich, St. Louis, MO, USA) according to the manufacturer’s recommendations. The quantity and integrity of total RNA were determined by a Thermo Scientific Nanodrop 2000 spectrophotometer. Complementary DNA (cDNA) was generated from 2 μg RNA using random primers and M-MLV Reverse transcription (Promega, Madison, WI, USA). Gene expression was assessed with 50 ng of cDNA using Custom TaqMan^®®^ Array 96-Well Fast Plates (Life Technologies, Thermo Fisher Scientific, Waltham, MA, USA) in an Applied Biosystems 7500 thermal cycler according to the manufacturer’s recommendations. Expression data were normalized using the 2^−ΔΔCt^ method with 18S, GAPDH, GUSB, and HRPT as endogenous reference genes.

### 2.8. Western Blotting

Protein extraction was performed according to our previous work [46]. Briefly, cell lines and primary cultures were lysed using a radioimmunoprecipitation assay (RIPA) lysis buffer containing phosphatase inhibitors (PhosSTOP^TM^, Roche, CHE) and protein inhibitors (cOmplete^TM^ Mini EDTA-free, Roche, CHE) plus a general metalloprotease inhibitor, BB-94 10 µM. Proteins were purified by centrifugation at 12,000× *g* at 4 °C for 15 min and were, subsequently, quantified. After that, 20 μg of proteins was separated by electrophoresis on a 4–20% polyacrylamide gel electrophoresis (GenScript, Piscataway, NJ, USA) under denaturing and reducing conditions and then, was transferred to a nitrocellulose membrane (Thermo Scientific, MA, USA) at 350 mA for 1 h. Later, membranes were incubated with sodium citrate solution 0.01 M, pH 6.0, for 10 min at 95 °C in a water bath to expose the antigens. Next, membranes were blocked with a solution of 3% (*w*/*v*) bovine serum albumin 0.1% (*v*/*v*) Tween in Tris-buffered saline, pH 7.4, and incubated overnight with the respective primary antibodies. Finally, a second incubation took place with their respective secondary antibodies conjugated with horseradish peroxidase (KPL, Gaithersburg, MD, USA) diluted 1:5000 in a blocking solution for 1 h at room temperature. Peroxidase activity was detected by enhanced chemiluminescence (Pierce Biotechnology, Rockford, IL, USA), and the bands were quantified by densitometry using Image Studio Lite (LI-COR Biosciences, NE, USA).

### 2.9. Statistical Analysis

To evaluate if data were normally distributed, the Kolmorov–Smirnov normality of Graphpad Prism 5 test (Graphpad Software, San Diego, CA, USA) was used. Data were statistically analyzed by one-way ANOVA along with Dunnett’s post hoc test. Statistical significance was established at *p* < 0.05. The sample size (*n*) was 3 per group. We calculated it using G*Power software with the following parameters: effect size 1.5, alpha 0.05, and beta 0.9.

## 3. Results

### 3.1. E4 Effects in Proliferation, Apoptosis and Migration

**Figure 1:**
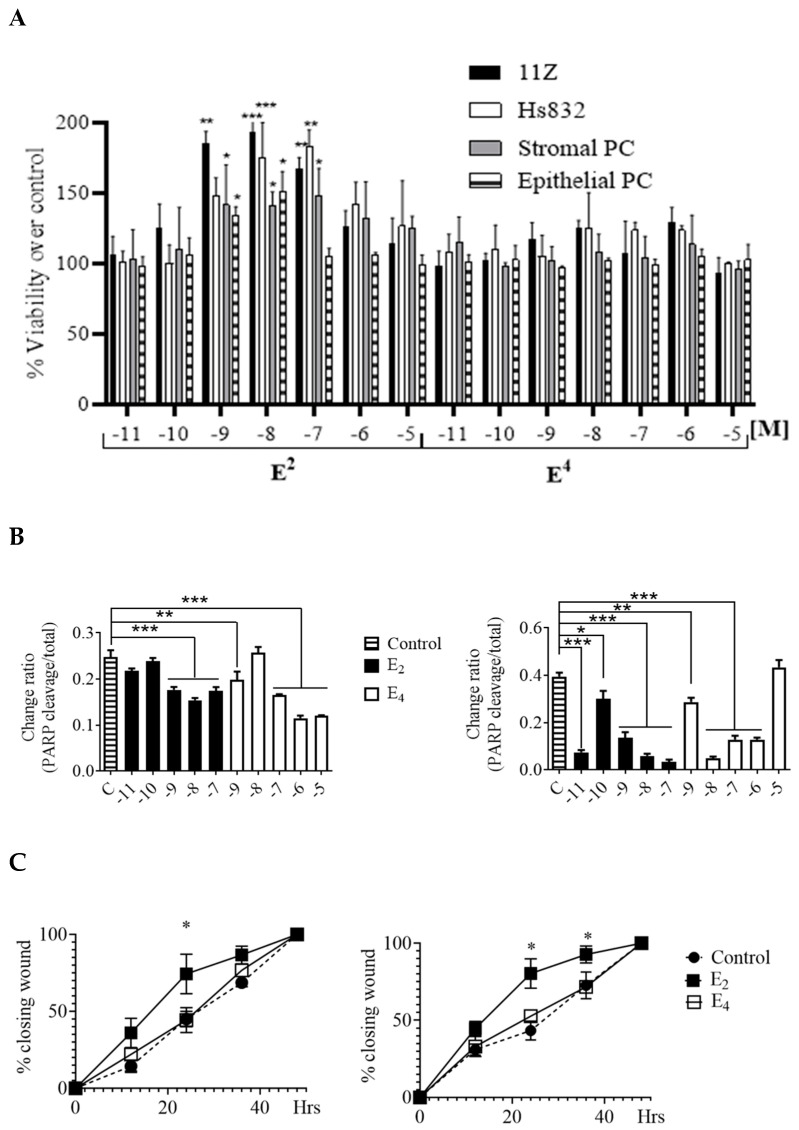
Effect of E4 on cell viability and PARP cleavage levels. (**A**) MTS analysis for endometriotic cell lines (11Z and Hs832) and stromal primary culture from patients. (**B**) Western blot analysis of PARP cleavage levels and (**C**) Scratch assay for 11Z cells (left) and Hs832 (right). Analyzed by one-way ANOVA along with Dunnett’s post hoc test. *n* = 3; * *p* < 0.05; ** *p* < 0.01; *** *p* < 0.001.

Considering the importance of estrogenic proliferation in endometriosis, we measured the cell viability by MTS under seven different concentrations (10^−11^ to 10^−5^ M) of E2 or E4 during 24 h (Figure 1A). As we expected, endometriotic cells increased their viability in the presence of E2 between 10^−9^ to 10^−7^ M for the cells studied. Despite being an estrogen, E4 did not induce significant changes in cell viability for any condition tested; this was observed for all cells treated. Regarding apoptosis (Figure 1B), it was measured by PARP cleavage ratio. Hs832 and 11Z cell lines were incubated at physiological and supra-physiological concentrations of E2 [43] and E4 [44] separately.

Both hormones showed a similar and consistent pattern of PARP cleavage ratio reduction. For epithelial 11Z cells, apoptosis was reduced at most concentrations of E4 and E2 (Figure 1B, left). For stromal cell line Hs832, a significant decrease was only observed for two conditions (10^−9/−7^ M) of E2, and almost (except 10^−8^ M) all E4 concentrations (Figure 1B, right). Migration, a crucial step for lesion establishment, was assessed by wound assay with E2 and E4 at physiological concentrations (10^−9^ and 10^−6^ M, respectively, Figure 1C). For both cell lines, E2 significantly increased migration (11Z: 24 h and Hs832: 24 h and 36 h, Figure 1C). Again, E4 did not show differences in respect to control at any studied time.

### 3.2. E4 Increases ERα/ERβ Ratio in Endometriotic Cell Lines

**Figure 2:**
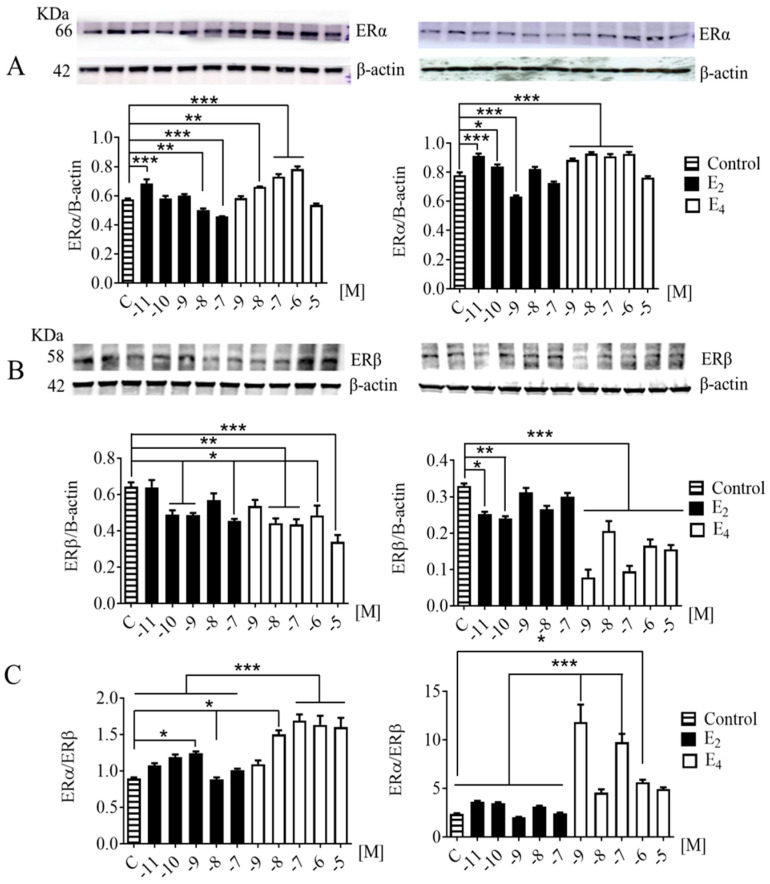
ERs protein levels measurement by Western blot after E2 or E4 treatments in endometriotic cells. (**A**) ERα levels over β-actin levels (AU) for 11Z cells (left) and Hs832 (right). (**B**) ERβ levels over β-actin levels (AU) for 11Z cells (left) and Hs832 (right). (**C**) ERα/ ERβ rate values for 11Z cells (left) and Hs832 cells (right). (*n* = 3), * *p* < 0.05, ** *p* < 0.01, *** *p* < 0.001, by one-way ANOVA along with Dunnett’s post hoc test.

Considering the role of ERs in endometriosis, we evaluated their levels after E2 or E4 incubation in epithelial and stromal cells. For ERα, E2 administration resulted in spread results, showing increased (11Z: 10^−11^ M; Hs832: 10^−11/−10^ M, Figure 2A) and decreased levels in both cell lines (11Z: 10^−8/−7^ M; Hs832: 10^−9^ M, Figure 2A).

Interestingly, incubation with E4 showed a solid increment in ERα levels in both cell lines at most concentrations used (Figure 2B). Regarding ERβ levels, there was a significant decrease after E2 incubation at some conditions (11Z: 10^−10/−9/−7^ M; Hs832: 10^−11/−10^ M, Figure 2B). For E4, reduced ERβ protein levels were observed at most concentrations used for 11Z and all conditions for Hs832 cells (Figure 2B). Finally, in Figure 2C, E4 significantly increased ERα/ERβ ratio with respect to control and above all E2 conditions; this was observed at 10^−7/−6/−5^ M for 11Z cells and at 10^−9/−7^ M for Hs832 cells. For E2, the ERα/ERβ ratio was increased only in epithelial cells at10^−7^ M.

### 3.3. E4 Increases P4 in Endometriotic Cell Lines

**Figure 3:**
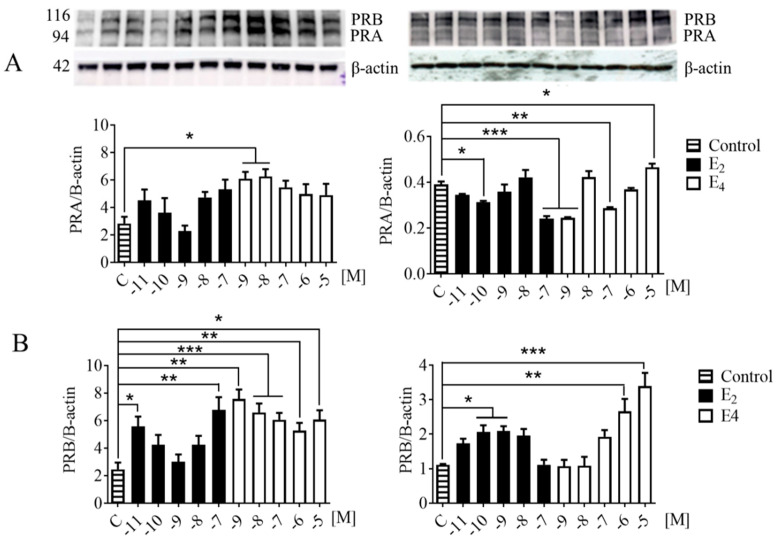
PRs protein levels measurement by Western blot after E2 or E4 treatments in endometriotic cells. (**A**) PRA levels over β-actin levels (AU) for 11Z cells (left) and Hs832 (right). (**B**) PRB levels over β-actin levels (AU) for 11Z cells (left) and Hs832 (right) (*n* = 3), * *p* < 0.05, ** *p* < 0.01, *** *p* < 0.001, by one-way ANOVA along with Dunnett’s post hoc test.

An evaluation of PRs showed a spread response for PRA according to conditions and cell lines. Incubation with E2 only showed a significant decrease in protein levels for Hs832 cells at 10^−10/−7^ M. In the case of E4 incubation, an increase in PRA protein levels was induced (11Z: 10^−9/−8^ M; Hs832: 10^−5^ M, Figure 3A), but a decrease was also observed in two conditions (Hs832: 10^−9/−7^ M, Figure 3A). For PRB protein levels, incubation with E2 resulted in increased levels at some concentrations (11Z: 10^−11/−7^ M: Hs832: 10^−10/−9^ M, Figure 3B). For E4, a more consistent increment was observed for all concentrations used in 11Z cells and at 10^−6/−5^ M for Hs832.

### 3.4. E4 Effects in Primary Culture Protein Levels

**Figure 4:**
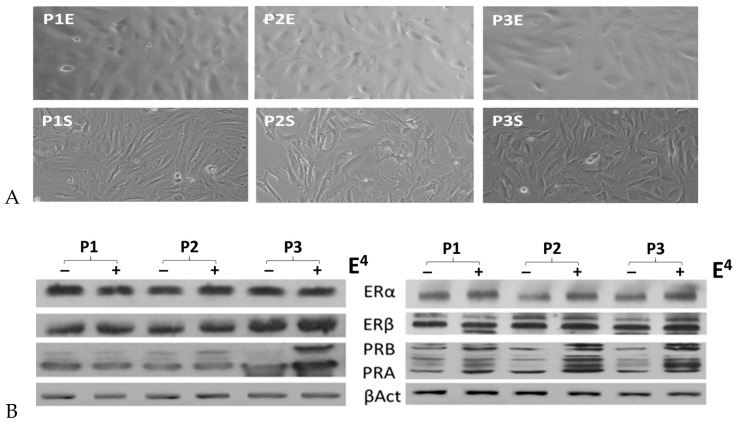
E4 effect in endometrial primary culture cells. (**A**) 20× images of primary cultures, (top) epithelial cells (bottom) stromal cells. (**B**) Western blot analysis of primary culture cells. (left) epithelial cells (right) stromal cells.

We evaluated the effect of E4 in endometrial primary culture cells, which were obtained from endometriotic patients. Our protocol allowed us to separate epithelial from stromal cells, both of which could grow and proliferate under our conditions. Epithelial cells showed a characteristic shape with rounded borders, and stromal cells looked elongated and smaller (Figure 4A). There was no difference among samples, nor in shape variation after treatment. Later, we measured the protein levels of ERs and PRs for these cells treated with 10^−6^ M of E4 during 24 h. Epithelial cells (Figure 4B, left) seemed to maintain ERα and ERβ levels unchanged. Regarding PRB levels, there was an increment for patients 2 and 3, whereas PRA increased clearly for patient 3. On the other hand, stromal cells (Figure 4B, right) were showed to increase ERα, PRB, and PRA levels in all patients after E4 treatment. The protein levels of ERβ remained similar for both conditions.

### 3.5. E4 Increases P4 Response in Cell Lines

**Figure 5:**
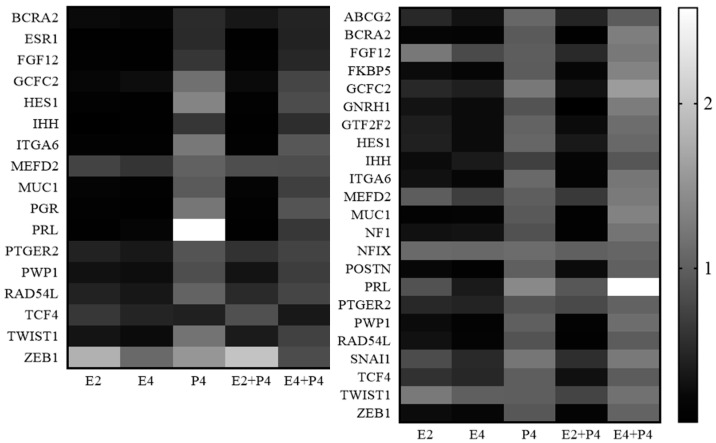
Heatmap of Progesterone receptor pathway RT-qPCR array analysis. Endometriotic cell lines 11Z (**left**) and Hs832 (**right**) were incubated under the following conditions: vehicle 24 h; E2 24 h; E4 24 h; P4 6 h; E2 24 h + P4 6 h; and E4 24 h + P4 6 h. Results are expressed as 2-ddCT.

Considering that after E4 incubation, PRs levels were consistently increased in endometriotic cell lines and primary cultures, we decided to evaluate if this is accompanied with an increased progesterone response by measuring expression levels of 28 genes related to progesterone response in human endometrium [47]. Considering our previous results (Figure 3), E2 and E4 incubation was performed during 24 h. Later, we added P4 at physiological concentration (10^−7^ M) according to [48] for 6 h. Genes with undetermined CT values for any of the conditions used were excluded from the analysis, giving a total of 17 genes for 11Z and 23 for Hs832 cells. The heat map (Figure 5) showed that P4 had higher expression values compared to E2 and E4 treatments (15 for 11Z and 23 for Hs832). Additionally, the combination of E4+P4 showed a higher expression compared to E2+P4 treatment (15 of a total of 17 genes). For stromal Hs832 cells, E4+P4 condition showed higher expression values than P4 alone treatment (18 of 23 genes).

## 4. Discussion

The present work showed the effects of E4 in endometriotic cells, comparing epithelial and stromal type under the influence of E4 or E2. First, we noticed that E4 did not induce proliferation or migration in these cells, which represents an important finding, considering that estrogens are the leading molecule for endometriosis, an estrogen-dependent disease. Moreover, it raised the levels of PRs, which boosts the response to progesterone. The association between PRs levels and P4 response is a crucial first step, suggesting that PRs induced by E4 are functional proteins that elicit activation of PRs-related genes, required to restore P4 response in patients who developed resistance or prevent its appearance [25,33]. Interesting differences arose from Figure 1, where incubation with E2 increased cell number and reduced apoptosis. For both results, the data described an unimodal curve, a characteristic trend in E2 response, that was reported for endometriotic cells previously [18]. However, E4 did not change cell viability at any concentration. A possible explanation is that in our in vitro model, proliferation was increased by E2 but not affected by E4; therefore, cells remained in a constant number even if apoptosis was reduced. E4 incubation did not induce a significant variation in these events (cell growth and migration, Figure 1C) in accordance with authors that considered E4 as a “weak” estrogen compound, in tissues such as the uterus, breasts, and vessels [2,7,12,13,49]. This represents an interesting feature for using E4 in endometriosis treatment, where estrogenic effects are associated with disease progression. Nevertheless, our in vitro studies separately analyzed epithelial and stromal cells; so, different effects might arise in in vivo models and also to different endometriosis entities [50].

An estrogenic response is a crucial event for endometriotic progression, as it is not only determined by E2 concentration but also to ERs levels [36,37,38,39,40]. A significant increase in ERα or reduction in ERβ levels can be considered as a beneficial effect for endometriosis treatment [5,15,26,37]. A proper measurement of both variables is ERα/ERβ ratio, which is chronically reduced in endometriosis [36,40,51]. Incubation with E4 was highly effective, elevating ERα/ERβ ratio, by increasing ERα and decreasing ERβ protein levels at the same time. None of the E2 conditions showed this behavior (Figure 2C).

Regarding PRs levels, PRA is known to act as a repressor of PRB [52,53], but it is uncertain if the reduction observed in Hs832 cells (Figure 3A) was associated with a stronger P4 response. For epithelial cells, E4 showed a more solid PRs increment compared to E2, whereas for stromal cells, there was not a clear trend. Nevertheless, PRB levels were clearly elevated by E4, as this receptor was directly linked to progesterone therapeutic effects in endometriosis [54].

Under our conditions, the comparison between E2 and E4 revealed interesting advantages for the natural hormone in focus of endometriosis treatment, namely: the absence of proliferation or migration, and increased ERα/ERβ ratio and PRs levels. These features highlight the major problems of endometriosis treatment, high estrogenic response and diminished sensitivity to progesterone. Nevertheless, it was necessary to determine if E4 could replicate these effects in other cellular models. For this reason, we performed primary cultures from endometriotic patients who did not receive hormonal treatment to avoid the long term effects of steroids on these cells. Primary cultures were performed successfully, but the number of cells obtained were not enough to replicate all experiments; therefore, proliferation and steroid receptors levels were chosen according to relevance and feasibility. However, this model could replicate the results for proliferation (Figure 1A), for both cell types, and steroid receptor levels for stromal cells (Figure 4), which were previously observed for cell lines. This may imply that those epithelial cells do not respond to E4, or they require interaction with stromal during their response to estrogens, which was reported previously [55].

Among this study’s strengths, we highlighted that E4, an estrogenic molecule, does not elicit proliferation or migration, which favors disease spread. Additionally, there was a consistent association between the increment in PR protein values and P4 response, which validates the methodology. However, there are limitations that we would like to establish. In vitro assay results only sometimes correlate with in vivo scenarios. The reason for this is that in vitro assays do not allow cell interaction when new metabolites secreted by these cells can elicit a plethora of responses. Additionally, in vitro assays do not consider the interaction with other cells, such as the immune system or the metabolites that the liver releases during drug metabolization. In particular, the human uterus is a highly variable tissue, from physiological to pathological. In the case of endometriosis, different studies suggested that the environment changes through disease progression, from immunological components which lead to inflammation processes from the beginning of the disease to fibrotic activity at the late phases [56]. It is still uncertain about elucidating if E4 will be used for treatment and how it will affect the local inflammatory process. Secondly, many theories about endometriosis originate from classical retrograde menstruation, migration of bone marrow stem cells, hormonal induction, and environmental and epigenetics [57]. It is unclear if every endometriotic lesion will respond to treatment similarly, and this may implicate a different origin from the disease. Cell lines and primary cultures vary based on genetic variability among patients and the origin of endometriotic disease. For future in vivo studies of E4 in endometriosis models, it is possible that the effects shown in the present work will not arise or might be eclipsed by novel interactions between epithelial and stromal cells, which were treated separately in this study.

## 5. Conclusions

In conclusion, compared to E2, E4 showed exciting differences, such as not promoting proliferation or migration in all cellular models used. Moreover, the increment of PRs levels correlated with a more robust genetic response over the P4 stimulus. Both responses are significant for endometriosis treatment; first, proliferation and migration are common estrogenic effects in this tissue and are related to disease spread. Second, P4 resistance due to the reduction in PRs is the leading cause of recurrence. However, there were limitations to this study which require evaluating E4 effects in more complex models and also incorporating new variables such as inflammation, immune system, and disease origin, among others.

## Data Availability

Not applicable.
